# Maternal Mortality: A National Institutes of Health Pathways to Prevention Panel Report

**DOI:** 10.1097/AOG.0000000000005488

**Published:** 2023-12-21

**Authors:** Karina W. Davidson, Mary Beth Terry, Paula Braveman, Pamela J. Reis, Stefan Timmermans, John W. Epling

**Affiliations:** Northwell Health, Feinstein Institutes for Medical Research, Institute of Health System Science, New Hyde Park, and the Columbia University Mailman School of Public Health, Herbert Irving Comprehensive Cancer Center, New York, New York; the University of California, San Francisco, San Francisco, and the University of California, Los Angeles, Los Angeles, California; the East Carolina University College of Nursing, Greenville, North Carolina; and the Carilion Clinic and the Virginia Tech Carilion School of Medicine, Roanoke, Virginia.

## Abstract

The National Institutes of Health’s (NIH) Pathways to Prevention panel on postpartum health provides a consensus statement on the evidence, research gaps, and future priorities to prevent maternal morbidity and mortality. The panel reviewed an NIH-commissioned evidence review and workshop that included epidemiologic studies, demonstration interventions, and other maternal morbidity and mortality research to create these national recommendations. The panel concludes that a maternal morbidity and mortality crisis reflects a systemic failure of current U.S. health care, research efforts, and social policies. The panel recommends improving maternal health through a “maternal morbidity and mortality prevention moonshot” that adopts a comprehensive, multilevel life course conceptual framework; strengthens the research methods used within the science of maternal health; establishes and conducts national prevention, treatment, and policy interventions; and reimburses evidence-informed clinical approaches to improve maternal health across the life course. Without a national focus on fundamentally transformative interventions and other initiatives aimed at redressing structural racism and inequities in health care, current interventions and clinical advances in maternal morbidity and mortality prevention will remain tragically insufficient.

## Abstract 2

A federally funded moonshot initiative with resources, commitment, and equity focus is needed to transform maternal health research, health services, and policies to reduce mortality.

The United States remains an outlier among high-income countries in high health care spending and has the highest maternal mortality rates. The human toll of maternal mortality is devastating, with a national maternal mortality rate in the United States of 35 deaths per 100,000 women of reproductive age. This represents a daily toll of about 3.8 maternal deaths, a staggering number that omits the lifelong and profound toll on families and communities. The National Institutes of Health (NIH) cosponsored a 3-day workshop^1^ (Pathways to Prevention Program: Identifying Risks and Interventions to Optimize Postpartum Health, November 29–December 1, 2022) to address these alarming statistics and to develop potential proposed solutions. The NIH also commissioned an evidence review^2^ of the social (including structural) risk factors associated with maternal morbidity and mortality in the United States during the prenatal and postpartum periods. This consensus statement summarizes the panel's proposed conceptual framework and national blueprint for research and the transformative change needed to prevent maternal morbidity and mortality.

## MATERNAL MORBIDITY AND MORTALITY IN THE UNITED STATES

**Fig. 1.**
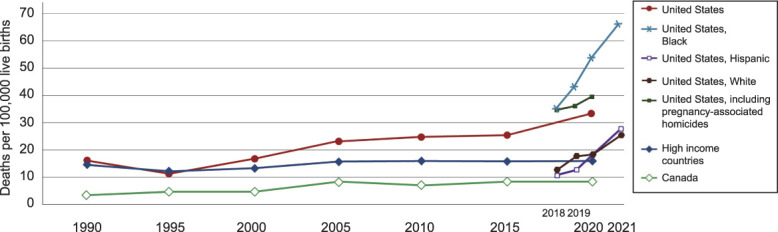
Maternal mortality rates in the United States, Canada, and all high-income countries. Data from 2021 provisional.^9,36–38^

In 2019, the cost of maternal morbidity (ie, the sum of the cost of the most common nine health care conditions) was estimated to be at least $32.3 billion.^3^ Moreover, U.S. maternal mortality rates have increased markedly since the 1990s, whereas those of other high-income countries have decreased (Fig. 1). The United States has experienced major increases in maternal mortality rates every year for the past several decades across all racial and ethnic groups. These average increases hide racial inequities with rates that are about two to three times higher in Black, Native American, and Alaska Native^4^ individuals in the United States compared with White individuals. Individuals identifying as Hispanic have rates similar to or lower than those of White individuals.^5^ Provisional data for 2021 show that absolute rates are increasing across all racial and ethnic subgroups, but the higher relative ratios in Black, Native American, and Alaska Native people persist, translating into a much larger absolute increase in deaths for these racial and ethnic groups. The overall pattern shows that year-to-year increases are not attributable to a single infectious disease but rather to chronic systems failures that affect the most vulnerable.^6,7^

*Davidson. Preventable Maternal Mortality. Obstet Gynecol 2024*.

**Fig. 2.**
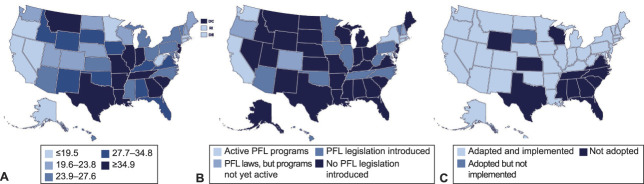
Maternal mortality (**A**), Paid Family Leave (PFL) laws (**B**), and Medicaid expansion by state (**C**).^39–41^

Many upstream social factors in the United States may in turn explain these maternal mortality rate inequities. For example, the United States is the only high-income country that does not provide guaranteed paid parental leave and provides variable postpartum care, creating considerable economic barriers that may contribute in part to continued maternal mortality inequities among different racial and ethnic subgroups.^8^ Figure 2 shows the major geographic differences in maternal mortality by state (Fig. 2A), the states that have enacted Paid Family Leave (Fig. 2B), and the states that have expanded access to postpartum care through Medicaid expansion (Fig. 2C). The striking contrasts across states reveal major inequities in health, based on where one lives, and support the potential of enacting federal policies to help prevent maternal mortality.

*Davidson. Preventable Maternal Mortality. Obstet Gynecol 2024.*

Our understanding of maternal mortality suffers from gaps in surveillance and duration of that surveillance. For example, although violence is an important cause of maternal morbidity and mortality, intimate partner violence rates and homicide rates are not accurately reported in perinatal and postpartum individuals; thus, neither is included in national rates.^9^ Figure 1 demonstrates the further increase in overall maternal mortality rates when homicide rates are included in national statistics.

Fifty-two percent of maternal deaths occur after delivery and within the first year after birth.^1^ However, the definition of maternal mortality typically is restricted to the period during pregnancy and up to 42 days after delivery.^1^ This arbitrary time restriction prevents a complete picture of morbidity and mortality outcomes and inequities associated with pregnancy. Therefore, in this report, maternal morbidity and mortality denote pregnancy-related health conditions and deaths up to 1 year after childbirth.

## CONSENSUS PANEL DATA SOURCES

The panel drew on two evidence sources to create its consensus report: a commissioned evidence-based review of the literature and a 3-day NIH workshop. First, the Minnesota Evidence-Based Practice Center, under contract with the Agency for Healthcare Research and Quality, conducted an evidence review^2^ and prepared a report summarizing existing research on the social (including structural) risk factors associated with maternal morbidity and mortality in the United States during the prenatal and postpartum periods, which is published as a companion piece in this issue.^10^ The commissioned evidence review searched for epidemiologic studies examining social (including structural) risks associated with maternal morbidity and mortality. Social risks or determinants of health are “the conditions in the environments where people are born, live, learn, work, play, worship, and age that affect a wide range of health, functioning, and quality of life outcomes and risks.”^11^ They include poverty, racism, and lack of education. The reviewers classified studies according to research design and rigor of the analytic approach, including an assessment of study bias. Inclusion criteria, key questions, and search strategies are provided in the companion commissioned review.^10^ Although the reviewers identified 8,378 unique references, only 1.4% (n=118 studies) were deemed eligible on the basis of the prespecified inclusion criteria. These 109 studies were all observational, and the review found that all were at risk for more than minimal bias. Most studies also did not address the interaction or intersectionality of multiple risk factors. Many studies focused on violence and trauma, with fewer studies focusing on other social risks such as identity and discrimination, socioeconomic factors, psychological stress, racism (including structural and institutional racism), and environmental factors. The commissioned review did not assess interventions.

Second, the NIH sponsored a 3-day workshop (recording of presentations can be found at https://prevention.nih.gov/research-priorities/research-needs-and-gaps/pathways-prevention/identifying-risks-and-interventions-optimize-postpartum-health) that included speakers conducting research and demonstration projects to address the burden of maternal morbidity and mortality in the United States. The workshop presenters provided a selective review of prevalence, prevention, interventions, and current research efforts to address maternal morbidity and mortality. They provided examples of the types of research and local demonstration projects that address severe maternal morbidity (eg, severe bleeding, infection, preeclampsia and eclampsia, complications from unsafe abortion, and chronic mental health and cardiovascular conditions) and maternal mortality, including homicide. Finally, an independent panel of individuals from multiple disciplines reviewed the commissioned review and the findings presented at the workshop to propose the transformative change needed to prevent maternal morbidity and mortality.

## CONSENSUS REPORT CONCLUSIONS AND RECOMMENDATIONS

The commissioned review reported that most of the otherwise eligible studies in this field do not meet the usual minimal risk of bias quality filter applied in systematic reviews. This could be attributable to unique challenges, methodologic issues, or a lack of available high-quality studies in the field. The panel concluded that a high-priority need for future studies is that they are designed to have a low risk of bias.

The workshop reported research and demonstration or intervention projects focused primarily on single levels (eg, a focus on clinicians only or reimbursement only) rather than on multiple levels and sectors in the same project. The panel concluded that although individually promising, the projects reported in the workshop are insufficient to reverse current maternal morbidity and mortality trends.

After considering the commissioned review and workshop presentations, the panel is convinced of the urgent need for a more comprehensive and fundamental change in approach to both research and national interventions addressing maternal morbidity and mortality. The panel concluded that this new approach needs to tackle the root causes of maternal morbidity and mortality rates and implement effective multilevel solutions across the nation, not just in selected communities or states. This will require a different level of research, policy, and medical reimbursement funding. It will also require complex conceptual frameworks if transformative change is going to be accomplished. Five recommendations were developed that are based on these considerations (Appendix 1, available online at http://links.lww.com/AOG/D536, has detailed recommendations).

### Comprehensive Multilevel Life Course Conceptual Framework for Policy, Research, and Clinical Transformations

**Fig. 3.**
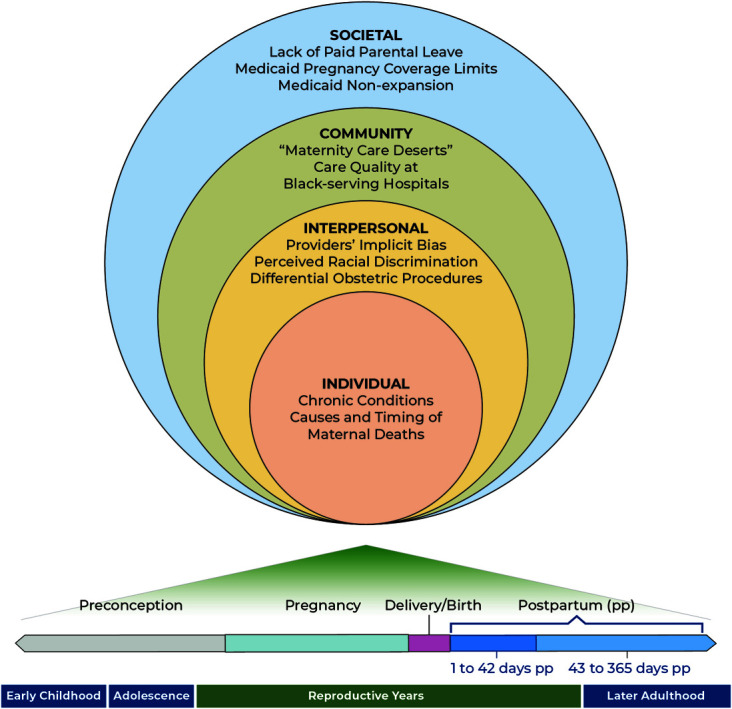
Example of one multilevel life course conceptual framework from ecologic systems theory. Adapted with permission from Noursi S, Saluja B, Richey L. Using the ecological systems theory to understand Black/White disparities in maternal morbidity and mortality in the United States. J Racial Ethn Health Disparities 2021;8:661–9. doi: 10.1007/s40615-020-00825-4^12^

First, to improve the quality and relevance of research on maternal mortality, a multilevel life course conceptual framework is needed. This new paradigm for both practice and systems innovation and research must address the social determinants of maternal mortality across an individual's life course and the immediate social and health care needs during the pregnancy and postpartum periods. An example of a multilevel life course conceptual framework is presented in Figure 3.^12^ Such a framework needs to recognize that societal and community factors, as well as interpersonal ones, affect maternal outcomes.

*Davidson. Preventable Maternal Mortality. Obstet Gynecol 2024.*

A new life course framework would explicitly acknowledge that maternal morbidity and mortality are affected by social (ie, nonmedical) determinants of health, including the upstream structural determinants of health, and that these exist at the individual, interpersonal, community, and societal levels and across the life course. These structural or systemic determinants are, by definition, the most fundamental determinants of health. They include racial residential segregation,^13^ voter suppression, lack of paid health insurance or family leave, and biased lending, policing, and sentencing. They are structures and systems such as laws, policies, and deeply entrenched practices and beliefs; they include what Dawes^14^ has aptly called “the political determinants of health.” Through many causal pathways and physiologic mechanisms, structural determinants initiate causal chains, ultimately resulting in ill health for disenfranchised, marginalized, and excluded groups of people, for example, groups defined by race and ethnicity,^13,15^ economic resources, sexual orientation, gender identity, and other characteristics historically associated with discrimination.^16^ Structural racism is a structural determinant that compounds the adverse effects of inadequate health care for communities of color and is one of the root causes of excess maternal mortality for people of color.^17,18^ Structural racism influences maternal morbidity and mortality in many complex ways. For example, residential segregation traps people of color in areas of low economic opportunity, often exposing them to environmental hazards, the placement of which has been driven by environmental injustice.^13^ When structural racism takes the form of voter suppression or gerrymandering, it results in communities of color lacking representation throughout the policy-making and resource allocation processes that affect so many aspects of life and health.^15,16^ All of these and other examples of structural racism can influence maternal health in myriad ways.^18^ Without application of a conceptual framework that includes structural determinants, excess maternal morbidity and mortality rates will continue unaddressed.

The life course approach holds that health and experiences at each life stage influence future health.^19^ A life course conceptual framework draws attention to reproductive health trajectories, reproductive choices across the lifespan, developmental effects of early childhood experiences, the importance of critical or sensitive periods, and the cumulative effects of chronic stressors (eg, the weathering hypothesis regarding the creation and exacerbation of racial disparities by racism-related chronic stressors).^20,21^ Such a framework should incorporate the contributions of family, friends, neighbors, community groups, public health organizations, and conditions outside of health—such as economic security, housing, education, and nutrition—that powerfully shape health to address the multiple risk factors that result from social and structural determinants of health. The framework should also consistently apply a “disparities lens” to studies—always examining how indicators vary over time across more and less socially advantaged groups of people.^15,16^ Applications of this type of conceptual framework to research have been accomplished by the Center for Translation Research and Implementation Science at the National Heart, Lung, and Blood Institute, and methodology workshop material is available for those considering designing such research (https://www.nhlbi.nih.gov/events/2015/multi-level-intervention-research-methods-recommendations-targeting-hard-reach-high).

### Strengthen the Research Methods Used in the Science of Maternal Health

Second, addressing maternal morbidity and mortality systematically will require improving methods, data sets, causal impact analyses, risk of bias, and other research methods. Given the scope of the problem and the multilevel and life course influences on maternal morbidity and mortality, these research methods must boldly address the problem using systems thinking. Research into a complex, systemic health problem calls for innovative research methods and creativity in designing interventions that affect all levels of the multilevel life course model. Examples of such innovative research methods and projects can be seen in the Agency for Healthcare Research and Quality–funded learning laboratory grants.^22^

Prevention science should invest substantially in studies of new research methods to improve studies of the quality of clinical and equitable care, particularly for those who have experienced the greatest disparities. It requires studying the social (including structural) determinants of maternal health in health care and public health, as well as across all societal sectors and contextual factors. To support these strengthened research methods, consensus on standardized reporting of outcomes, collection of data sets, and inclusion of pregnant and postpartum individuals from diverse backgrounds and life experiences in studies must be accomplished. Federal funding agencies will be needed to lead the creation of these standardized outcomes and methods and then fund these proposed activities.

### Establish Programs of Research to Conduct Needed Prevention and Treatment Studies to Guide Efforts to Reduce Maternal Morbidity and Mortality

Intervention studies are particularly needed now. Orienting federal research funding agencies and research agendas toward a focus on prevention and treatment intervention will help focus on what is needed to address the challenges in maternal health and their drivers. Given the importance of the life course approach, these intervention studies should focus on longer-term outcomes and capture key potential determinants of health. They should be informed by a systematic review of existing interventions; therefore, some of these grant opportunities should consider 8-, 10-, or even 15-year timeframes. The Diabetes Prevention Program^23^ had a 10-year follow-up that demonstrated that the delay in development of diabetes seen during the intervention was sustained.^24^ Longer-term follow-up demonstrated no mortality differences among the treatment arms, a critical finding for understanding whether delaying diabetes incidence improved longevity.^25^ Without longer timeframes, changes in mortality cannot be ascertained.

### Pay for Evidence-Based Clinical Approaches and Train the Needed Workforce

To ensure that research findings can be put into practice immediately, access to services should be expanded through Medicaid expansion, and coverage should be mandated for maternal, newborn, and mental health services for up to 1 year.^26,27^ Clinician shortages in underserved communities should be addressed with innovative programs and workforces when such programs and workforces have been developed, evaluated for improving outcomes, and found to be evidence based.^28,29^ Federal agencies should fund training and educational programs to expand the workforce needed to deliver these new interventions. To accomplish the above four recommendations, we propose a final action.

### A Maternal Morbidity and Mortality Prevention Moonshot

Finally, the panel concluded that a transformative level of national commitment to address maternal morbidity and mortality is required if any lasting change is expected to occur. In light of the persistent and increasing socioeconomic and racial health disparities in the United States, advances in maternal health will inevitably fall short if the conditions in the places where individuals are born, live, learn, work, and age put their health at risk. Addressing health disparities requires dismantling social inequities in a broad range of institutions. Effective prevention strategies also need to consider the injury-related causes of maternal mortality such as substance overdose, homicide, and suicide. Addressing maternal morbidity and mortality, therefore, likely requires strengthening the social safety net to address both these precursors and consequences that endanger pregnant individuals.^30^ Improving maternal health requires a holistic solution that reflects patients' and frontline health care professionals' experiences and addresses persistent social inequities in health outcomes. This includes addressing a person's substance use disorders, mental health issues, level of education, childhood trauma, and many other aspects of whole-person care that have been missing from our approach to maternal care. Improving perinatal health outcomes requires a holistic perspective, crossing boundaries between health care, federal and state policies, innovative multilevel and life-course informed research, and social services. This will require dismantling disciplinary and sectoral silos. Programs such as Paid Family Leave, universal health insurance, universal childcare, and investment in “communities of opportunity” will need to be rigorously tested to see whether they provide a benefit in reducing maternal morbidity and mortality.

How do we get there? A “moonshot” is a bold effort to achieve a potentially impossible task.^31^ Although current NIH funding focusing on maternal health outcomes has been increasing (eg, $224 million in 2020),^32^ it still is a tiny fraction of the resources mobilized for the original moonshot of the U.S. Apollo space program 60 years ago. There is precedent for a moonshot initiative to address health care problems. In 2016, U.S. Congress passed the 21st Century Cures Act, providing about $2 billion in research funding for what was called the Cancer Moonshot.^33,34^ In February 2022, President Biden revived the Cancer Moonshot program and set new and ambitious goals. The Cancer Moonshot has positively affected cancer prevention and care innovations, health inequities, structural barriers to health, partnerships, and funding—many of the same areas that need to be addressed in maternal morbidity and mortality.

A similar moonshot approach is needed to address maternal morbidity and mortality.^35^ This moonshot must establish annual goals and clear objective metrics to be monitored at the national level to ensure progress rather than a continuation of the worsening of outcomes in pregnant individuals. During the past 3 decades, as the U.S. maternal mortality rate has been increasing, the overall global rate of maternal mortality has declined dramatically. Other high-income countries have maternal mortality rates one third to one half of the U.S. rate. Calling for many practical and structural policy-related efforts does not need to wait for further advancements in basic science or incremental health care delivery and access improvements. Instead, with the use of implementation science focused on interventions, creating a National Maternal Learning Health System that identifies, prioritizes, funds, and conducts high-quality, rigorous national demonstration projects needs to start now. Our recommendation for a maternal mortality moonshot is a 50% or more reduction in preventable maternal mortality and an elimination of racial and ethnic disparities over the next 10 years.

We have seen multiple examples of small, isolated, single-level, local prevention interventions and programs that have shown significant promise to address maternal mortality if brought to scale.^10^ The five recommendations above can be fulfilled only with a different approach. A moonshot initiative is required to scale these programs and to properly evaluate their implementation. With resources, commitment, and a focus on equity in the areas of prevention, public health, research, and health care access and quality, we can reduce maternal death and equitably promote maternal health.
